# Comparison of plasma ALZpath p-Tau217 with Lilly p-Tau217 and p-Tau181 in a neuropathological cohort

**DOI:** 10.1186/s40478-025-02064-2

**Published:** 2025-06-30

**Authors:** Divya Bali, Gemma Salvadó, Thomas G. Beach, Geidy E. Serrano, Alireza Atri, Eric M. Reiman, Andreas Jeromin, Oskar Hansson, Shorena Janelidze

**Affiliations:** 1 Clinical Memory Research Unit, Department of Clinical Sciences, Lund University, Sölvegatan 19, BMC B11, Lund, 22184 Sweden; 2 Civin Laboratory for Neuropathology, Banner Sun Health Research Institute, 10515 W Santa Fe Drive, Sun City, Arizona 85351 USA; 3 Cleo Roberts Clinical Center, Banner Sun Health Research Institute, 10515 W Santa Fe Drive, Sun City, Arizona 85351 USA; 4 Banner Alzheimer’s Institute, Phoenix, AZ USA; 5 Department of Psychiatry, University of Arizona College of Medicine, Phoenix, Arizona USA; 6 Arizona Alzheimer’s Consortium, Phoenix, AZ USA; 7 ASU-Banner Neurodegenerative Disease Research Center, Arizona State University, Tempe, AZ USA; 8 Atlantic Biomarkers, LLC, Alachua, FL USA; 9 Eli Lilly and Company, Indianapolis, IN USA

**Keywords:** Alzheimer’s disease, Phosphorylated-tau, Amyloid plaques, Neurofibrillary changes

## Abstract

There is an urgent need for accurate and validated methods to measure plasma phosphorylated tau (p-Tau) biomarkers in Alzheimer’s disease (AD). In this study, we compared the performance of new commercially available plasma p-Tau217 assay developed by ALZpath to other established p-Tau assays in an autopsy-confirmed sample. We included 72 participants from the Arizona Study of Aging and Neurodegenerative Disorders cohort, with antemortem plasma assessments and postmortem neuropathological examination. Plasma p-Tau217 was measured using the single molecule arrays (SIMOA)-based ALZpath immunoassay and plasma p-Tau217 and p-Tau181 were analyzed with mesoscale discovery (MSD)-based immunoassays developed by Lilly Research Laboratories. Plasma biomarkers were compared with densities of post-mortem-assessed amyloid plaques and neurofibrillary changes. Levels of p-Tau217_ALZpath_, p-Tau217_Lilly_ and p-Tau181_Lilly_ were significantly associated with plaque (partial Spearman ρ = 0.58, ρ = 0.78, ρ = 0.65; *p* < 0.001) and neurofibrillary (ρ = 0.26, ρ = 0.51, ρ = 0.37; *p* < 0.05) density scores, adjusting for age, sex, and time of interval between blood sampling and death. All three biomarkers were also significantly associated with plaque density scores (p-Tau217_ALZpath_, ρ = 0.53; p-Tau217_Lilly_, ρ = 0.73; p-Tau181_Lilly_, ρ = 0.59; *p* < 0.001), when additionally adjusting for neurofibrillary density scores. However, only p-Tau217_Lilly_ was significantly associated with neurofibrillary density scores (ρ = 0.32, *p* = 0.022), when adjusting for plaque density scores. The correlations of p-Tau217_ALZpath_ and p-Tau181_Lilly_ with plaque density scores were comparable, whereas p-Tau217_Lilly_ exhibited significantly higher correlations with plaques (p_diff_≤0.015) and neurofibrillary changes (p_diff_≤0.004) than p-Tau217_ALZpath_. While p-Tau217_ALZpath_ and p-Tau181_Lilly_ predicted the presence of Alzheimer’s disease neuropathological change (ADNC), Braak staging (Braak 0-IV vs. Braak V-VI) and CERAD neuritic plaque classification (low/sparse vs. moderate/frequent) with similar accuracies (area under the curve [AUC]_range_, 0.74–0.79), p-Tau217_Lilly_ AUCs were significantly higher (AUC_range_,0.82–0.89, p_diff_≤0.024) than the AUCs of p-Tau217_ALZpath_. In conclusion, p-Tau217_ALZpath_ exhibited similar performance as p-Tau181_Lilly_ but its correlations with core measures of AD pathology were significantly lower in comparison with p-Tau217_Lilly_. Future studies are warranted to replicate these findings in larger independent cohorts.

**Supplementary Information:**

The online version contains supplementary material available at 10.1186/s40478-025-02064-2.

## Introduction

With recent breakthrough advancements in the field of Alzheimer’s disease (AD), anti-amyloid immunotherapy treatments have been approved by the Food and Drug Administration (FDA) and have made their way from the laboratory workbench to the bedside of the patients [1–5]. Implementation of cost-effective, scalable blood tests to identify patients eligible for such treatments in routine clinical practice as well as for screening and monitoring treatment effect in clinical trials may significantly reduce economic and patient burden. The advances in the identification and validation of plasma biomarkers have led to promising findings [6].

Blood phosphorylated tau (p-Tau) has been shown to accurately reflect AD-associated amyloid and tau pathologies as well as distinguish AD dementia from non-AD neurodegenerative diseases [7–9]. Over the years, various blood p-Tau variants (i.e. p-Tau217, p-Tau181, p-Tau231, p-Tau205, p-Tau202) [7, 8]^10^– [12] have been measured but among those variants, p-Tau217 has emerged as one of the most promising blood-based biomarkers (BBMs) of AD [8, 13–15]. Notably, plasma p-Tau217 demonstrated a larger dynamic range and higher performance against core AD biomarkers such as cerebrospinal fluid (CSF), PET imaging, and clinical and neuropathology outcomes [8, 11]^16^– [19]. Plasma p-Tau217 levels measured by Lilly and Janssen platforms, have shown higher diagnostic and prognostic accuracy than p-Tau181 measures in several in vivo studies [13, 20, 21]. Recently, ALZpath has developed an immunoassay based on Single molecule array (SIMOA) platform for measuring p-Tau217 in blood as well as cerebrospinal fluid (CSF). This assay has been commercially available for research purposes globally. Some recent studies have demonstrated a high clinical diagnostic accuracy of plasma p-Tau217_ALZpath_ in various cohorts [22–25]. But none of the studies have evaluated the performance of p-Tau217_ALZpath_ against the gold standard neuropathological outcomes. In a previous study, we found that both plasma p-Tau217 and p-Tau181 measured using Lilly immunoassays, had significant associations with postmortem density measures of plaques and tangles, with p-Tau217 showing stronger associations [26]. Here, we aimed to assess the ALZpath p-Tau217 immunoassay against neuropathological measures and compare its performance with other established p-Tau assays, i.e. p-Tau217_Lilly_ and p-Tau181 _Lilly_ based on mesoscale discovery (MSD) platform.

## Materials and methods

### Study participants

This study included samples obtained through autopsies of 72 participants from the Arizona Study of Aging and Neurodegenerative Disorders (AZSAND) and Brain and Body Donation Program (BBDP) at Banner Sun Health Research Institute [27]. This longitudinal clinicopathological study enrolls normal and neurologically impaired participants from metropolitan Phoenix, Arizona. The diagnosis of participants included in this study varied from Cognitively unimpaired (CU) to mild cognitively impairment (MCI), AD and non-AD dementia patients. Detailed information about the recruitment process and inclusion/exclusion criteria has been previously described [27].

### Plasma analysis

**Fig. 1:**
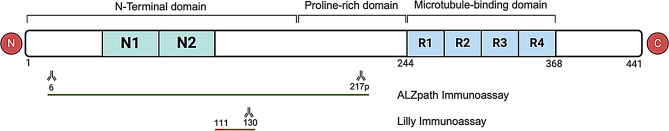
A schematic overview of the p-Tau assays. Illustration of full-length tau, including N-terminal domain, proline rich domain and microtubule binding domain. The binding sites for antibodies are indicated under the respective epitope region for both assays (ALZpath and Lilly). The image was created using BioRender

Plasma p-Tau217 concentration was measured at ALZpath, in Phoenix, Arizona, using the ALZpath immunoassay on the SIMOA platform as previously described [22]. Plasma p-Tau217 and p-Tau181 were analyzed at Lund University, Lund, Sweden, using the Lilly immunoassays on the Mesoscale discovery (MSD) according to the published protocols [28–30]. For ALZpath immunoassay, a proprietary monoclonal p-Tau217 specific antibody, an N-terminal detector antibody and a peptide calibrator were used (Fig. 1) [22]. For the Lilly immunoassays (Fig. 1), the small-spot streptavidin coated MSD plate was incubated with 25 µl of 0.5 µg/ml anti-p-Tau217 capture antibody (biotinylated-IBA493) and 1 µg/ml anti-p-Tau181 capture antibody (biotinylated-IB406) per well for 1 hour. Then, 50 µl of calibrators and samples (diluted 1:1 in a sample buffer) were added to each plate and incubated for 2 hour. Finally, the plate was incubated with 25 µl of 0.02 µg/ml anti-tau detection antibody (SULFO-TAG-4G10-E2) per well for 1 hour. All incubations were performed on a temperature-controlled shaker at 650 rpm. In the final step, the plate was read on the MSD SQ120 plate reader. The immunoassay was calibrated using synthetic p-Tau217 and p-Tau181 peptides respectively.

Plasma p-Tau217_Lilly_ and p-Tau181_Lilly_ data have been previously published as a part of a larger BBDP cohort [26] and are included in the present study only for comparison with p-Tau217_ALZpath_.

### Brain autopsy and neuropathologic assessments

The neuropathological examinations were carried out by a unique certified neuropathologist (TGB). The human brain tissue processing has been mentioned in detail previously [27]. The neuropathologist who performed the histopathological scoring was blinded to the neuropathological and clinical diagnosis. The density of amyloid plaques (both diffuse and neuritic) and neurofibrillary changes (neurofibrillary tangles and neuropil threads) were graded at standard sites in frontal, temporal, and parietal cortices as well as the hippocampus and entorhinal cortex as described previously [27]. In line with recommendations from a landmark paper by Braak & Braak, the dystrophic neurites within neuritic plaques were not included in the assessments of neurofibrillary changes because their occurrence is highly variable between subjects [31]. The total plaque density score was calculated using the published CERAD templates; first each region was rated as none, sparse, moderate, or frequent [32] and then converted into 0–3 scores in each region, which were then combined to give a total plaque score of maximum value 15. The total neurofibrillary density score was also calculated in the similar way using the CERAD templates. In addition, the topographical distribution of neurofibrillary changes were rated using Braak staging [31]. The CERAD neuritic plaque density score and Thal amyloid phase for Aβ plaque brain distribution were determined based on the published criteria [32, 33]. The global measure of Alzheimer’s disease neuropathologic change (ADNC) was obtained using the three global scales (Braak, CERAD and Thal) as described in the NIAA guidelines [34]. In some analyses, ADNC score was dichotomized as significant (if scores were intermediate or high) and non-significant (if scores were none or low) AD pathology.

### Statistical analysis

R studio (2022.12.0 + 353) and SPSS version 28 (IBM) were used to perform statistical analysis, and the data was visualized using R studio. Spearman’s correlation was used to determine the correlations among different plasma biomarkers (p-Tau217_ALZpath_ v/s p-Tau217_Lilly_ and p-Tau181_Lilly_ respectively). Partial Spearman’s rho (ρ) was used to assess the associations of p-Tau217 _ALZpath_ and p-Tau217_Lilly_ or p-Tau181_Lilly_ with amyloid plaque density scores or neurofibrillary density scores adjusting for co-variates including age, sex and time between blood drawn and death (time blood-death). In separate analysis, specific correlations of plasma biomarkers with plaque density scores were also adjusted for neurofibrillary density scores and vice versa. A bootstrapping approach (*n* = 1,000) was used to measure the significant differences between correlation coefficients. Independent associations between different plasma biomarkers and both amyloid plaque density scores and neurofibrillary density scores were measured as the percentage of partial Spearman ρ of amyloid/tau pathology over the sum of the partial Spearman ρ of the two pathologies (% partial ρ = 100*partial ρ/ (partial ρ_[plaque]_ + partial ρ_[neurofibrillary changes]_). The overall differences in the levels of different plasma biomarkers between pathological groups were assessed using the Kruskal-Wallis and Mann-Whitney U tests. The diagnostic accuracy of different plasma biomarkers was determined using receiver operating characteristic (ROC) curve analysis. The DeLong test was used to compare the area under the curve (AUC) of two ROC curves. P-values were corrected for multiple comparisons using Benjamini Hochberg’s false discovery rate; *p* < 0.05 was considered statistically significant.

## Results

### Participants

We included 72 participants from the Arizona Study of Aging and Neurodegenerative Disorders cohort with antemortem plasma samples and postmortem neuropathological examination (Table 1). The median age of study participants was 85 years, 29 (40.3%) were females and *APOE ε4* prevalence was 30.6%. Participants were categorized into two groups based on the ADNC scale [34]; none/low ADNC (*n* = 30, clinical diagnosis: 8 controls, 3 MCI, 19 non-AD neurodegenerative diseases) and intermediate/high ADNC (*n* = 42, clinical diagnosis: 7 controls, 6 MCI, 14 AD, 15 non-AD neurodegenerative diseases).

**Table 1:** Characteristics of study participants

	Total *n* = 72	None/low ADNC *n* = 30	Intermediate/high ADNC *n* = 42
Age, median [range]	85.4 [65.0,98.0]	86.0 [75.0, 98.0]	85.0 [65.0, 97.0]
Sex, women, n (%)	29 (40.3%)	11 (36.7%)	18 (42.9%)
*APOE*$\:\varvec{\epsilon\:}4$carriership, n (%)	22 (30.6%)	3 (10.0%)	19 (45.2%)
Time b/w blood sampling and death days, median [range]	3.16 [1.92, 54.2]	3.08 [2.25, 54.2]	3.21 [1.92, 7.57]
Plaque density scores, median [range] ^a^	9.50 [0, 15.0]	0 [0, 7.50]	13.8 [2.00, 15.0]
Neurofibrillary density scores, median [range] ^a^	6.75 [3.00, 15.0]	6.50 [3.00, 13.0]	7.00 [3.00, 15.0]
CERAD, n (%)			
Zero	18 (25.0%)	18 (60.0%)	0 (0%)
Sparse	14 (19.4%)	12 (40.0%)	2 (4.8%)
Moderate/Frequent	40 (55.6%)	0 (0%)	40 (95.2%)
Braak staging, n (%)			
Stage 0-IV	53 (73.6%)	26 (86.7%)	27 (64.3%)
Stage V-VI	19 (26.4%)	4 (13.3%)	15 (35.7%)
Plasma levels			
Plasma p-Tau217_ALZpath_, pg/ml	0.645 [0.10, 2.67]	0.430 [0.10, 2.57]	0.865 [0.23, 2.67]
Plasma p-Tau181_Lilly_, pg/ml	1.84 [0.73, 7.93]	1.43 [0.79, 4.94]	2.26 [0.73, 7.93]
Plasma p-Tau217_Lilly_, pg/ml	0.285 [0.03,1.60]	0.182 [0.03,0.502]	0.396 [0.06, 1.60]

Data are shown as median [range] unless otherwise specified. Participants were categorized into two groups based on ADNC (None/low vs. Intermediate/high) scale

^a^ Total density scores of amyloid plaques and neurofibrillary changes, respectively

Abbreviations: p-Tau, phosphorylated Tau

### Correlations between plasma biomarkers

**Fig. 2:**
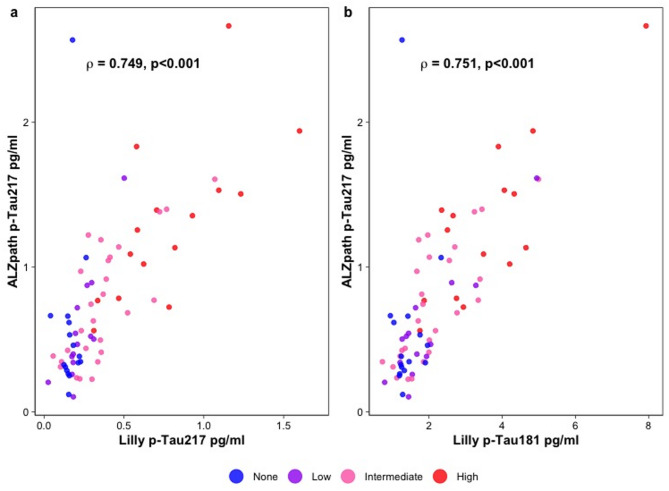
Correlation between different plasma biomarkers. Spearman’s correlation analysis was used to assess the association of p-Tau217_ALZpath_ with p-Tau217_Lilly_ (**a**) and p-Tau181_Lilly_ (**b**). Abbreviations: p-Tau, phosphorylated tau

We first assessed the association between p-Tau217_ALZpath_ vs. p-Tau217_Lilly_ and p-Tau181_Lilly_ using Spearman’s correlation (Fig. 2). In the whole cohort, p-Tau217_ALZpath_ was found to be significantly correlated with both p-Tau217_Lilly_ and p-Tau181_Lilly_ (ρ = 0.749 and ρ = 0.751, respectively; *p* < 0.001).

### Association of plasma biomarkers with plaque and neurofibrillary density scores

**Fig. 3:**
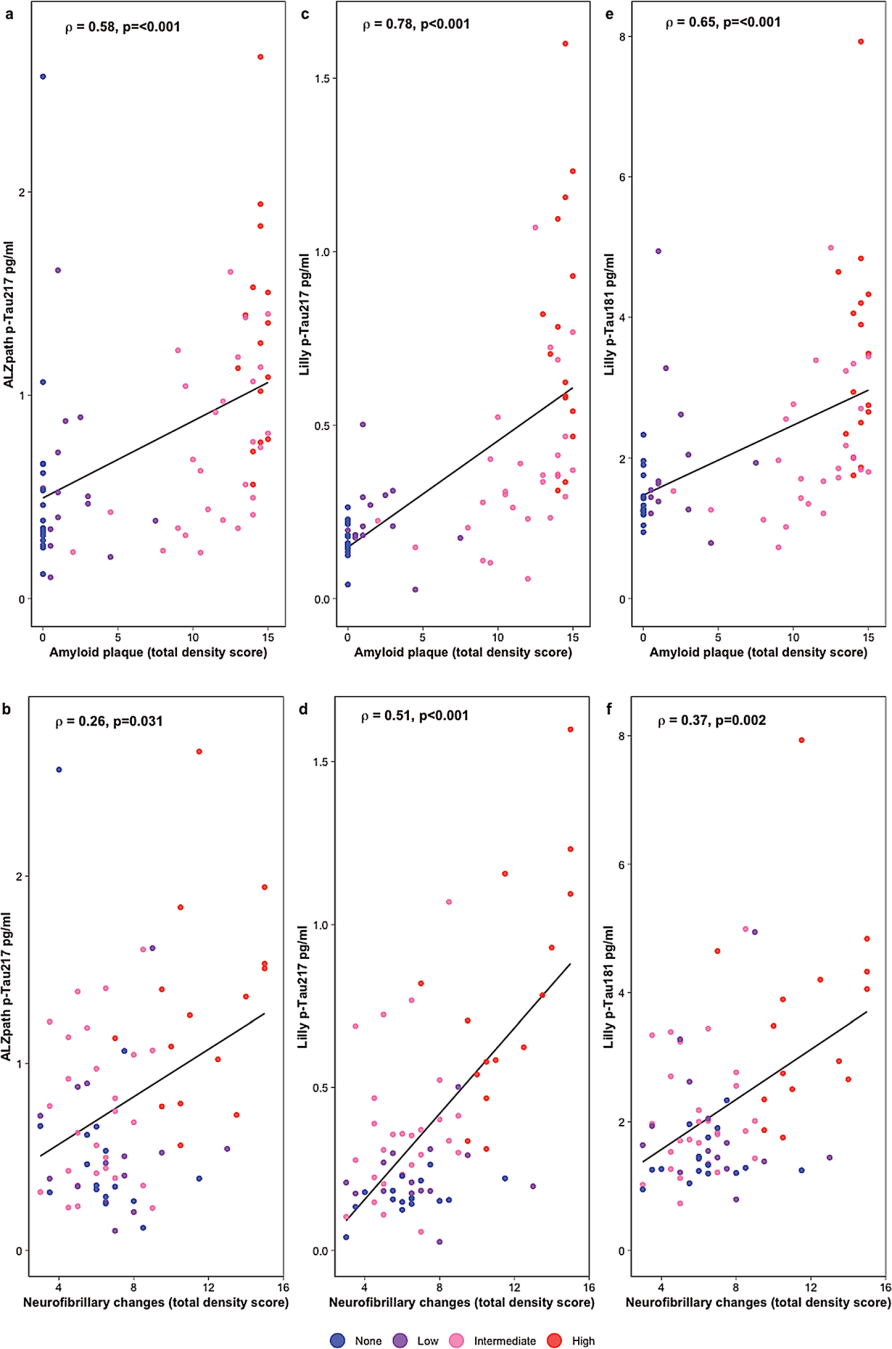
Association of p-Tau217_ALZpath,_ p-Tau217_Lilly_ and p-Tau181_Lilly_ with amyloid plaque density scores and neurofibrillary density scores. Association of p-Tau217_ALZpath_ (**a-b**), p-Tau217_Lilly_ (**c-d**) and p-Tau181_Lilly_ (**e-f**) with amyloid plaques (total density score) (**a**,** c**,** e**) and neurofibrillary changes (total density score) (**b**,** d**,** f**). Data are shown as partial Spearman correlation coefficients (ρ) and p-values. Plaque density scores and neurofibrillary density scores were measured on a semi-quantitative scale range from 0–3 using CERAD templates in five different brain regions and were added up to a total score range from 0–15. Data points are colored based on the ADNC classification. Abbreviations: ADNC, Alzheimer’s disease neuropathologic change; CERAD, Consortium to Establish a Registry for Alzheimer’s Disease; p-Tau, phosphorylated tau

We next investigated the association between different plasma biomarkers (p-Tau217_ALZpath_, p-Tau217_Lilly_ and p-Tau181_Lilly_) and measures of amyloid and tau pathologies, amyloid plaque density score and neurofibrillary density score, in separate models adjusted for age, sex and time blood-death (Fig. 3; Table 2). We found that all three plasma biomarkers were significantly associated with plaque density scores (p-Tau217_ALZpath_, ρ = 0.58; p-Tau217_Lilly_, ρ = 0.78; p-Tau181_Lilly_, ρ = 0.65; *p* < 0.001) as well as neurofibrillary density scores (p-Tau217_ALZpath_, ρ = 0.26, *p* = 0.031; p-Tau217_Lilly_, ρ = 0.51, *p* < 0.001; p-Tau181_Lilly_, ρ = 0.37, *p* = 0.002, Fig. 3). When examining the difference between the correlation coefficients, we found that p-Tau217_Lilly_ (but not p-Tau181_Lilly_) exhibited significantly higher correlations with both plaque density scores (ρ_diff_ = 0.20, *p* = 0.012) and neurofibrillary density scores (ρ_diff_ = 0.25, *p* = 0.004) than p-Tau217_ALZpath_.

**Table 2:** Association of p-Tau217_ALZpath_, p-Tau181_Lilly_ and p-Tau217_Lilly_ with amyloid plaque density scores and/or neurofibrillary density scores

	Models adjusted for covariates (age, sex and time blood-death)			Models adjusted for covariates and neurofibrillary density scores		
**Plaques**	𝝆	**p-value**	**p-value**	𝝆	**p-value**	**p-value**
p-Tau217_ALZpath_	0.58	**< 0.001**	Reference	0.53	**< 0.001**	Reference
p-Tau181_Lilly_	0.65	**< 0.001**	0.328	0.59	**< 0.001**	0.491
p-Tau217_Lilly_	0.78	**< 0.001**	0.012	0.73	**< 0.001**	0.015
	**Models adjusted for covariates (age**,** sex and time blood-death)**			**Models adjusted for covariates and plaques density scores**		
**Neurofibrillary changes**	𝝆	**p-value**	**p-value**	𝝆	**p-value**	**p-value**
p-Tau217_ALZpath_	0.26	**0.031**	Reference	0.03	0.82	Reference
p-Tau181_Lilly_	0.37	**0.002**	0.225	0.15	0.33	0.29
p-Tau217_Lilly_	0.51	**< 0.001**	0.004	0.32	**0.022**	0.003

Partial Spearman’s correlation (ρ) analysis was used to assess the association of p-Tau217_ALZpath_, p-Tau181_Lilly_ and p-Tau217_Lilly_ with amyloid plaque density scores or neurofibrillary density scores, adjusting for covariates including age, sex and time between blood drawn and death (time blood-death). In addition, correlations of plasma biomarkers with plaque density scores were also adjusted for neurofibrillary density scores and vice versa (last three columns). Significant associations (adjusted for multiple comparisons) are highlighted in bold. Bootstrapping was used to measure the significant differences between the correlation coefficients, using p-Tau217_ALZpath_ as reference

Abbreviations: p-Tau, phosphorylated tau

We found that all the three biomarkers were significantly associated with the plaque density scores when additionally adjusting for neurofibrillary density scores (p-Tau217_ALZpath_, ρ = 0.53, *p* < 0.001; p-Tau217_Lilly_, ρ = 0.73, *p* < 0.001; p-Tau181_Lilly_, ρ = 0.59, *p* < 0.001, Table 2). However, only p-Tau217_Lilly_ was significantly associated with neurofibrillary density scores when additionally adjusting for plaque density scores (ρ = 0.32, *p* = 0.022). Again, p-Tau217_Lilly_ had significantly higher correlation coefficients than p-Tau217_ALZpath_ (plaque density score: ρ_diff_ = 0.20, *p* = 0.015; neurofibrillary density score: ρ_diff_ = 0.29, *p* = 0.003).

**Fig. 4:**
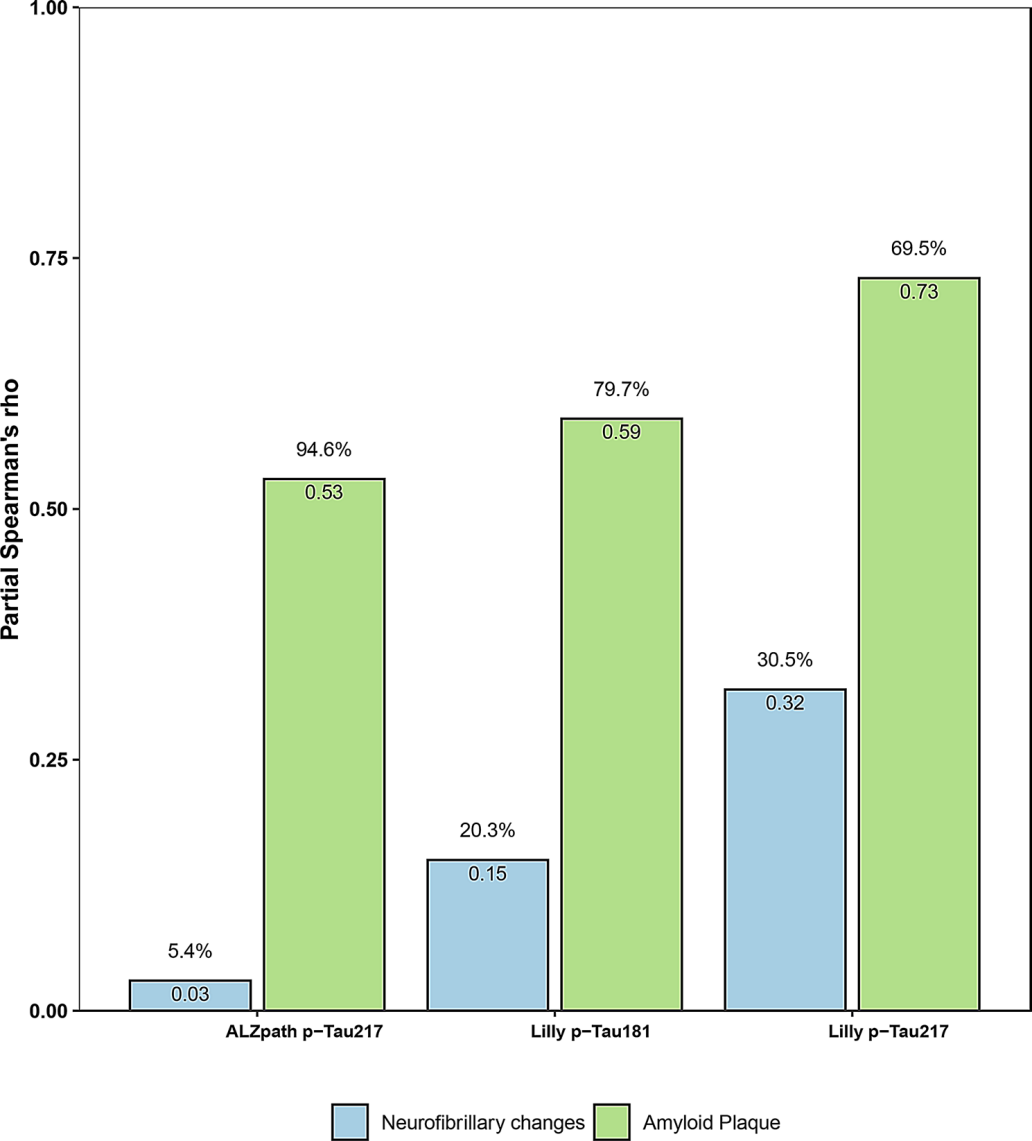
Independent associations between p-Tau217_ALZpath,_ p-Tau217_Lilly_ or p-Tau181_Lilly_ and both amyloid plaque density scores and neurofibrillary density scores. Correlations between plaque density scores and neurofibrillary density scores and p-Tau217_ALZpath,_ p-Tau217_Lilly_ or p-Tau181_Lilly_ after adjusting for other pathology load (i.e., when looking at neurofibrillary changes adjusting for plaques and conversely) as well as other covariates (age, sex and time between blood sampling and death). Partial Spearman’s rho (ρ) for each biomarker (p-Tau217_ALZpath_, p-Tau217_Lilly_ or p-Tau181_Lilly_) is indicated above the bars and percentual partial Spearman ρ over the sum of the partial Spearman ρ of the two pathologies (% partial ρ = 100*partial ρ/ (partial ρ_[plaque]_ + partial ρ_[neurofibrillary changes]_) is indicated below the bar. Abbreviations: p-Tau, phosphorylated tau

In addition, we tested the independent association between each plasma biomarker and both amyloid and tau pathologies, i.e., in the models including plasma biomarkers as outcome and both plaque density scores and neurofibrillary density scores as predictors (Fig. 4). These analyses revealed that the major proportion of variance in all three plasma biomarker levels was explained by plaques (p-Tau217_ALZpath_: 94.6%; p-Tau217_Lilly_: 69.5%; p-Tau181_Lilly_: 79.7%) than neurofibrillary changes (p-Tau217_ALZpath_: 5.4%; p-Tau217_Lilly_: 30.5%; p-Tau181_Lilly_: 20.3%).

### Association of plasma biomarkers with AD neuropathologic scales

**Fig. 5:**
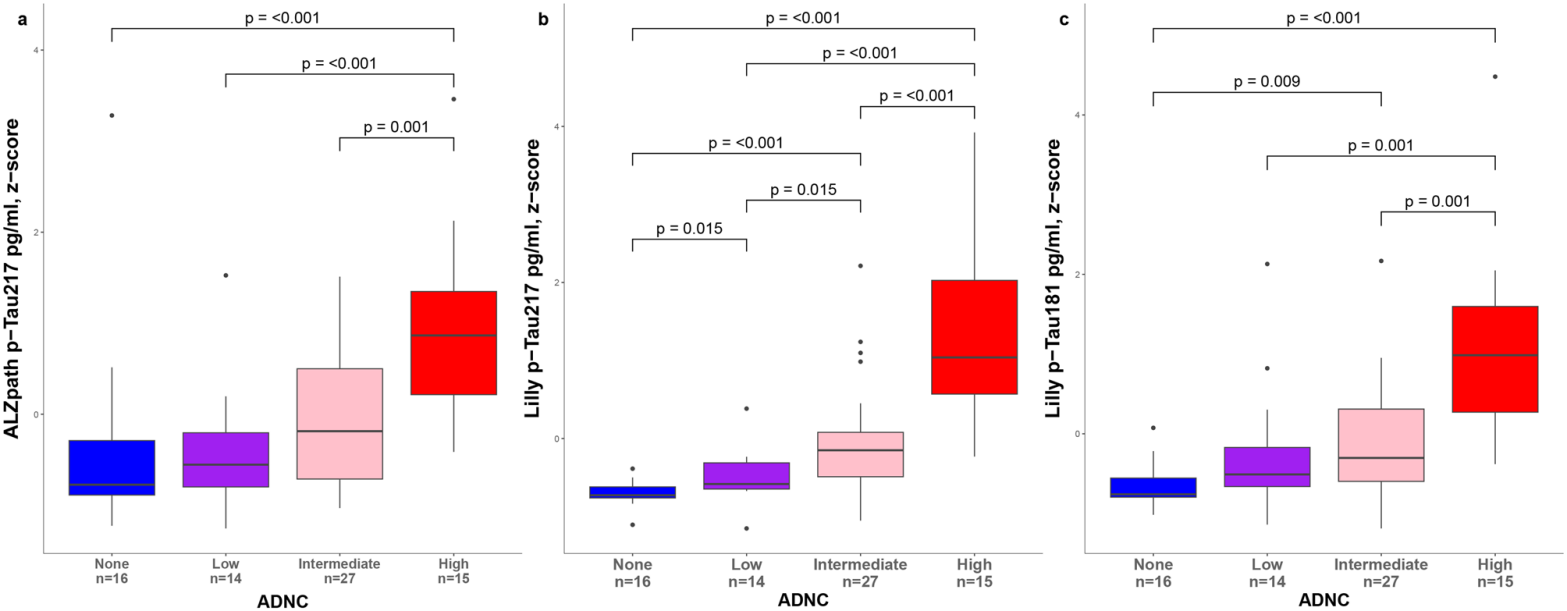
Levels of plasma biomarkers by ADNC classification. Plasma levels of p-Tau217_ALZpath_, (**a**), p-Tau217_Lilly_ (**b**) and p-Tau181_Lilly_ (**c**) by ADNC classification. Group differences were assessed using Kruskal Wallis test and Mann-Whitney U tests (p-values were corrected for multiple comparisons using FDR). Boxes show interquartile range, the horizontal lines are the medians, and the whiskers are plotted using Tukey method. Abbreviations: ADNC, Alzheimer’s disease neuropathologic change; FDR, false discovery rate; p-Tau, phosphorylated tau

We also examined the differences in plasma biomarker levels by the ADNC groups (none, low, intermediate, or high) (Fig. 5). P-Tau217_ALZpath_, p-Tau217_Lilly_ and p-Tau181_Lilly_ were all increased in high compared to none, low and intermediate ADNC (*p* ≤ 0.001). In addition, plasma levels of p-Tau217_Lilly_ (*p* < 0.001) and p-Tau181_Lilly_ (*p* = 0.009) but not p-Tau217_ALZpath_ were higher in the intermediate than none ADNC groups. P-Tau217_Lilly_ levels were also elevated in low vs. none (*p* = 0.015) and in intermediate vs. low (*p* = 0.015) ADNC. However, we did not find significant differences between these groups for p-Tau217_ALZpath_.

**Fig. 6:**
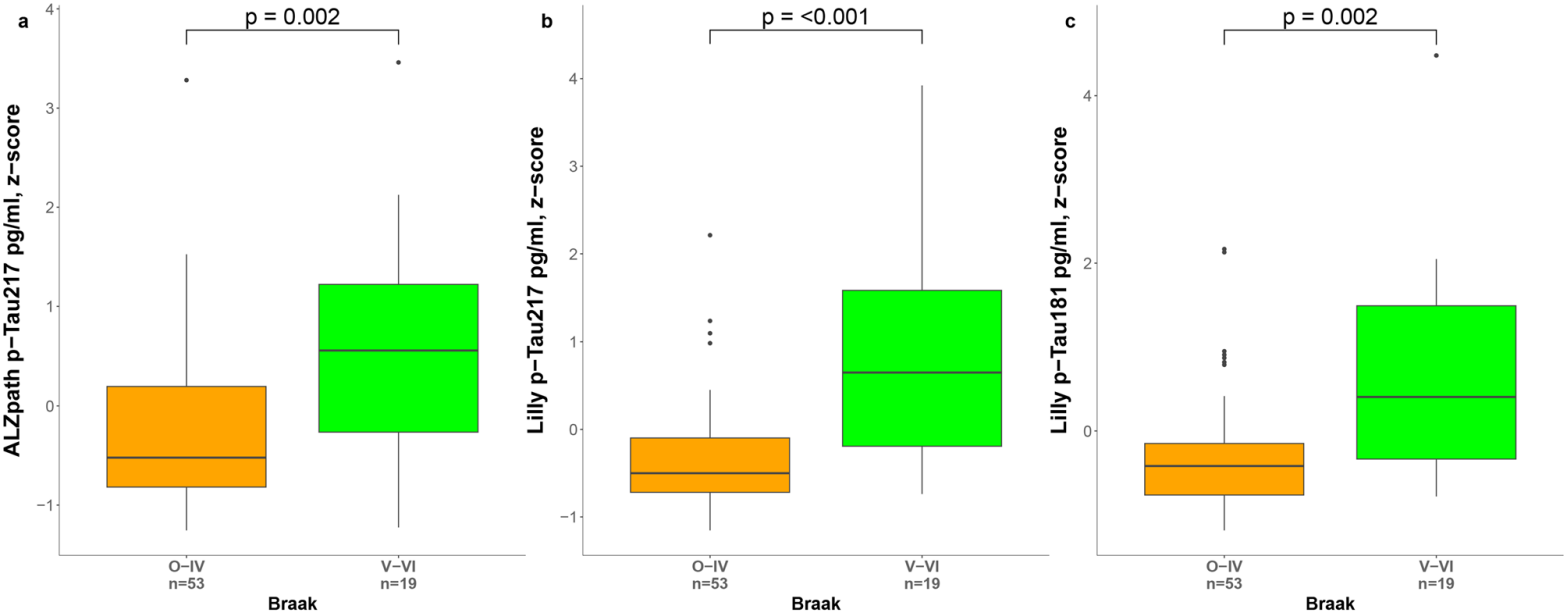
Levels of plasma biomarkers by Braak Staging. Plasma levels of p-Tau217_ALZpath_, (**a**), p-Tau217_Lilly_ (**b**) p-Tau181_Lilly_ (**c**) by Braak staging. Overall group differences were assessed using Mann-Whitney U tests (p-values were corrected for multiple comparisons using FDR). Boxes show interquartile range, the horizontal lines are the medians, and the whiskers are plotted using Tukey method. Abbreviations: p-Tau, phosphorylated tau

**Fig. 7:**
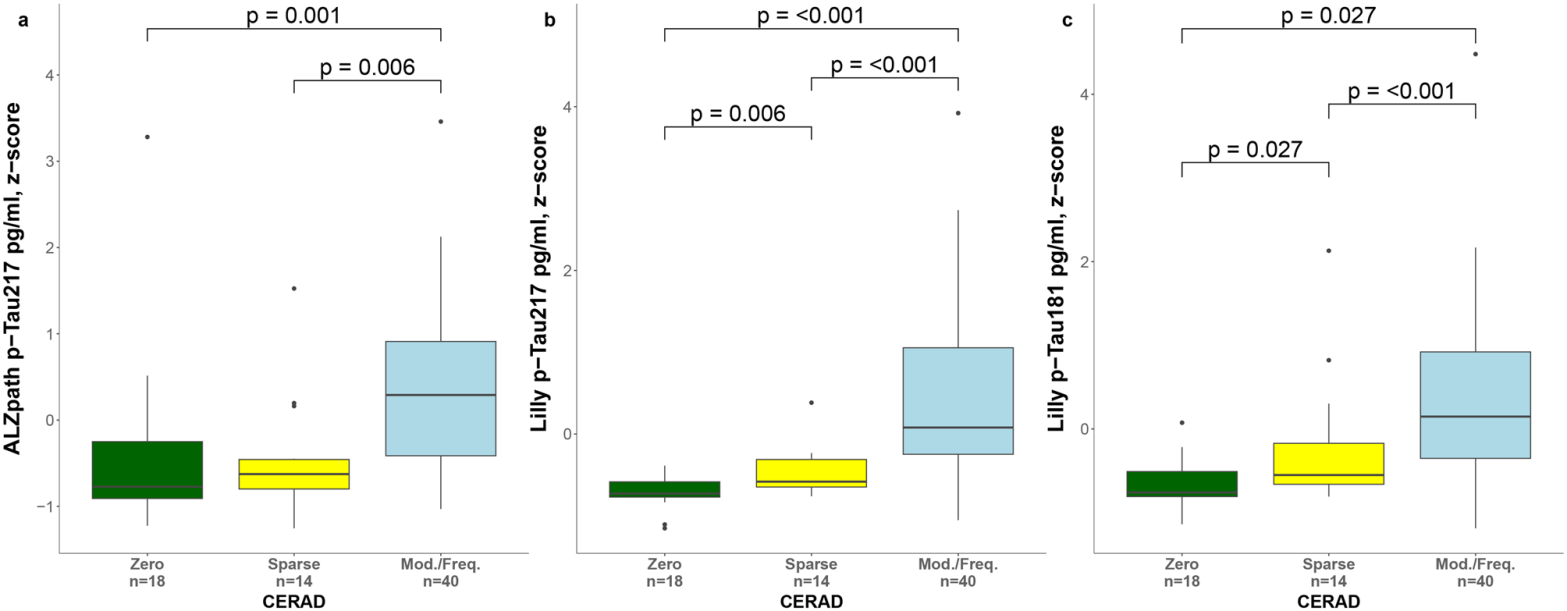
Levels of plasma biomarkers by CERAD classification. Plasma levels of p-Tau217_ALZpath_, (**a**), p-Tau217_Lilly_ (**b**) p-Tau181_Lilly_ (**c**) by CERAD classification. Group differences were assessed using Kruskal Wallis test and Mann-Whitney U tests (p-values were corrected for multiple comparisons using FDR). Boxes show interquartile range, the horizontal lines are the medians, and the whiskers are plotted using Tukey method. Abbreviations: CERAD, Consortium to Establish a Registry for Alzheimer’s Disease; FDR, false discovery rate; p-Tau, phosphorylated tau

Similarly, we investigated the differences in biomarker levels across global pathologic scales for neurofibrillary changes (Braak staging, Fig. 6) and neuritic plaques (CERAD classification, Fig. 7). Plasma levels of all three biomarkers were significantly lower in the Braak 0-IV group compared with the Braak V-VI group (p-Tau217_ALZpath_, *p* = 0.002; p-Tau217_Lilly_, *p* < 0.001; p-Tau181_Lilly_, *p* = 0.002). Furthermore, all the three biomarkers were increased in the moderate/frequent group in comparison to sparse and none groups on CERAD classification. However, only p-Tau217_Lilly_ and p-Tau181_Lilly_ showed significant differences between zero and sparse groups (p-Tau217_Lilly_, *p* = 0.006; p-Tau181_Lilly_, *p* = 0.027).

**Fig. 8:**
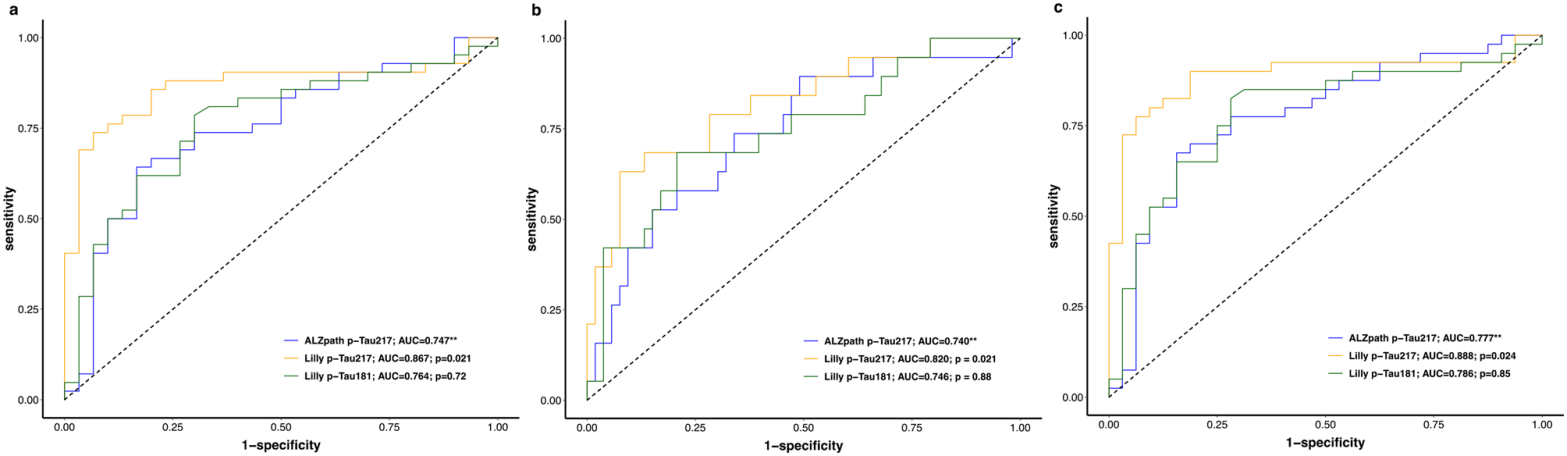
Predicting neuropathological scales classification. ROC curves analysis for predicting ADNC (**a**), Braak (**b**), and CERAD (**c**) classification. ADNC was dichotomized as none/low or intermediate/high, CERAD was dichotomized as low/sparse, or moderate/frequent and Braak stages were also dichotomized 0-IV or V-VI. The DeLong test was used to determine whether the area under the curve (AUC) of two ROC curves were significantly different (reference biomarker marked as (**); p-value < 0.05). Abbreviations: ADNC, Alzheimer’s disease neuropathologic change; AUC, Area under the curve; CERAD, Consortium to Establish a Registry for Alzheimer’s Disease; p-Tau, phosphorylated tau; ROC, receiver-operating characteristic

Additionally, we evaluated the accuracy of different plasma biomarkers to predict the presence of AD pathology, measured by ADNC (none/low vs. intermediate/high classification) (Fig. 8; Table S1). While all the three biomarkers were predictive of the presence of ADNC (AUC_range_, 0.75–0.87), p-Tau217_Lilly_ had significantly higher AUC (*p* = 0.021) in comparison to p-Tau217_ALZpath_. We performed similar set of analysis for Braak (0-IV vs. V-VI) and CERAD (low/sparse vs. moderate/frequent) classification (Fig. 8; Supplementary Table 1). Again, while all the three biomarkers were predictive of Braak staging and CERAD classification, p-Tau217_Lilly_ demonstrated significantly higher AUC compared to p-Tau217_ALZpath_ (Braak, p_diff_=0.021; CERAD, p_diff_=0.024). There were no significant differences in AUCs between p-Tau217_ALZpath_ and p-Tau181_Lilly_ for ADNC, Braak or CERAD neuritic plaque classifications.

## Discussion

The development of plasma biomarkers for AD has revolutionized the field. For instance, plasma p-Tau217 is already used in screening of participants in clinical trials and the Alzheimer’s Association has recommended to start implementation of BBMs including p-Tau217 in specialized memory clinics [6]. With such surging interest in p-Tau217, new p-Tau217 assays have been rapidly developed over the last several years. In this study, we assessed the performance of a novel commercially available p-Tau217_ALZpath_ immunoassay in relation to gold standard neuropathology and in comparison, to other established p-Tau assays i.e. p-Tau217_Lilly_ and p-Tau181_Lilly_. We found that p-Tau217_ALZpath_, p-Tau217_Lilly_ and p-Tau181_Lilly_ were associated with total amyloid plaque densities, with p-Tau217_Lilly_ (but not p-Tau181_Lilly_) showing significantly stronger correlations than p-Tau217_ALZpath_. At the same time, only p-Tau217_Lilly_ was significantly associated with neurofibrillary density scores when adjusting for amyloid plaque density scores. All three plasma biomarkers accurately predicted the presence of AD pathology, measured by NIA-AA ADNC as well as by Braak staging and CERAD neuritic plaque density, respectively. However, again p-Tau217_Lilly_ (but not p-Tau181_Lilly_) demonstrated significantly higher AUC compared to p-Tau217_ALZpath_. Taken together, our study provides evidence that p-Tau217_ALZpath_ and p-Tau181_Lilly_ exhibited similar performance whereas the association between p-Tau217_Lilly_ and core measures of AD pathology in this cohort were significantly stronger in comparison with p-Tau217_ALZpath_.

Previous studies have shown a statistically significant association of p-Tau217_ALZpath_ with in vivo CSF and PET measures of amyloid pathology and tau pathology [22, 23, 25]. In several cohorts including TRIAD, WRAP, SPIN, BALTAZAR and BioFINDER-2, p-Tau217_ALZpath_ has demonstrated a high fold-change in Aβ positive individuals compared with Aβ negative individuals, significant correlations with measures of amyloid PET, and has predicted with high accuracy abnormal Aβ status [22, 23, 25, 35]. Furthermore, in the TRIAD and BioFINDER cohorts, p-Tau217_ALZpath_ increased across Tau-PET defined Braak stages and correlated with tau-PET SUVR in temporal meta region [22, 25]. Likewise, in our study we also found that plasma p-Tau217_ALZpath_ levels correlated with postmortem amyloid density scores as well as neurofibrillary density scores. However, only p-Tau217_Lilly_ and not p-Tau217_ALZpath_ was associated with neurofibrillary density scores, when adjusting for amyloid density scores. Interestingly, in the in vivo Swedish BioFINDER-2 cohort, p-Tau217_Lilly_ was more strongly associated with tau-PET SUVR than p-Tau217_ALZpath_ whereas correlations with amyloid PET SUVR were similar for both biomarkers [25]. Altogether, our and previous results suggest that p-Tau217_ALZpath_ may have a stronger link association with amyloid pathology than tau pathology.

P-Tau217_Lilly_ outperformed p-Tau217_ALZpath_ for all neuropathological outcomes examined in the present study. In contrast, previous reports have indicated comparable performance of p-Tau217_ALZpath_ and other p-Tau217 assays such as p-Tau217_Lilly_, p-Tau217_Lumipulse_ and p-Tau217 + _Janssen_ when detecting positivity on Aβ-PET and tau-PET and differentiating AD from other neurodegenerative disease [24, 25, 36]. We speculate that these differences in results, could be related to sample characteristics, especially considering the high mean age of this cohort. Future head-to-head comparison studies are needed to assess the performance of p-Tau217_ALZpath_ to other assays in larger independent neuropathology cohorts.

To best of our knowledge, this is the first study to measure plasma p-Tau217 using the commercially available ALZpath immunoassay in a neuropathology cohort, which marks one of its main strengths. There were also some limitations to our study. First, the sample size was relatively small, but significant for a neuropathological cohort. Second, we used semiquantitative measures of amyloid and tau pathology which might have a reduced range as compared with continuous immunohistochemical measures, or in vivo Aβ-PET and tau-PET measures, although PET measures (which were not available in the BBDP cohort) are complicated by technical issues such as spill-in/out, and non-specific, off-target binding. Third, we only investigated correlations of plasma p-Tau biomarkers with neurofibrillary changes which included neurofibrillary tangles and neuropil threads. Further research is required to examine associations of plasma biomarkers with different types of AD-type tau pathology (e.g., neurofibrillary tangles, neuropil threads, dystrophic neurites within the neuritic plaques and those surrounding ghost tangles [37, 38]) separately as well as with other types of tau lesions, such as argyrophilic grains, astrocytic plaques, tufted astrocytes, coiled bodies and aging-related tau astrogliopathy (ARTAG). Future studies are also needed to compare the ability of different plasma p-Tau biomarkers to detect Aβ and tau pathologies in other non-AD neurodegenerative diseases [39, 40]. These studies would be of particular importance for evaluating the efficacy of novel AD targeting treatments in neurodegenerative diseases where AD co-pathology is frequently present.

In conclusion, our results indicate that plasma p-Tau217_ALZpath_ levels reflect abnormal accumulation of Aβ in the brain and are not significantly associated with neurofibrillary changes when adjusted for amyloid pathology. Furthermore, the performance of plasma p-Tau217_ALZpath_ in this cohort was similar to the performance of p-Tau181_Lilly_ but somewhat inferior as compared to p-Tau217_Lilly_. These findings will inform future studies on the utility of plasma p-Tau217 and its implementation in diagnostic workup of AD.

## Electronic supplementary material

Below is the link to the electronic supplementary material.

Supplementary Material 1

## Data Availability

Pseudonymized data will be shared by request from a qualified academic investigator for the sole purpose of replicating procedures and results presented in the article and as long as data transfer is in agreement with EU legislation on the general data protection regulation and decisions by the Swedish Ethical Review Authority and Region Skåne, which should be regulated in a material transfer agreement.

## Abbrevations

AD: Alzheimer’s disease
ADNC: Alzheimer´s disease neuropathological change
ARTAG: Aging-related tau astrogliopathy
AUC: Area under the curve
AZSAND: Arizona Study of Aging and Neurodegenerative Disorders
Aβ: Amyloid beta
BALTAZAR: Biomarker of AmyLoïd peptide and AlZheimer´s disease Risk
BBDP: Brain and Body Donation Program
BBMs: Blood-based biomarkers
CERAD: The Consortium to Establish a Registry for Alzheimer´s Disease
CSF: Cerebrospinal fluid
CU: Cognitively unimpaired
FDA: Food and Drug Administration
MCI: Mild cognitive impairment
MSD: Mesoscale discovery
NIA-AA: National Institute on Aging-Alzheimer´s Association
PET: Positron emission tomography
Phosphorylated tau: p-Tau
ROC: Receiver operating characteristic curve
SD: Standard deviation
Simoa: Single molecule assays
SPIN: The Sant Pau Initiative on Neurodegeneration
SUVR: Standard Uptake Value Ratio
TRIAD: Translational Biomarkers in Aging and Dementia
WRAP: Wisconsin Registry for Alzheimer´s Prevention
