# Supplementary material for: Comparison of plasma ALZpath p-Tau217 with Lilly p-Tau217 and p-Tau181 in a neuropathological cohort
Source: Acta Neuropathol Commun. 2025 Jun 30;13:144. doi: 10.1186/s40478-025-02064-2 (PMC12207803; doi:10.1186/s40478-025-02064-2)
Supplement: Supplementary file 1 — Supplementary Material 1 [file 40478_2025_2064_MOESM1_ESM.pdf]

Comparison of plasma ALZpath p-Tau217 with Lilly p-Tau217 and p-Tau181 in a neuropathological cohort

Divya Bali, Gemma Salvadó, PhD, Thomas G. Beach, MD, PhD , Geidy E. Serrano, PhD, Alireza Atri, MD, PhD, Eric M. Reiman, MD, Andreas Jeromin, PhD, Oskar Hansson, MD, PhD, Shorena Janelidze, PhD

Divya Bali, Clinical Memory Research Unit, Department of Clinical Sciences, Lund University, Lund 22184, Sweden; divya.bali@med.lu.se

Gemma Salvadó, Clinical Memory Research Unit, Department of Clinical Sciences, Lund University, Lund 22184, Sweden; gemma.salvado@med.lu.se

Thomas G. Beach, Civin Laboratory for Neuropathology, Banner Sun Health Research Institute, 10515 W Santa Fe Drive, Sun City, AZ 85351; thomas.beach@bannerhealth.com

Geidy E. Serrano, Civin Laboratory for Neuropathology, Banner Sun Health Research Institute, 10515 W Santa Fe Drive, Sun City, AZ 85351; geidy.serrano@bannerhealth.com

Alireza Atri, Cleo Roberts Clinical Center, Banner Sun Health Research Institute, 10515 W Santa Fe Drive, Sun City, AZ 85351; Alireza.atri@bannerhealth.com

Eric M. Reiman, Banner Alzheimer's Institute, Phoenix, Arizona, USA; Department of Psychiatry, University of Arizona College of Medicine, Phoenix, Arizona, USA; Arizona Alzheimer's Consortium, Phoenix, Arizona, USA; ASU-Banner Neurodegenerative Research Center, Arizona State University, Phoenix, Arizona, USA; Neurogenomics Division, Translational Genomics Research Institute, Phoenix, Arizona, USAanner Alzheimer's Institute, Phoenix, AZ, 85006, USA; Eric.Reiman@bannerhealth.com

Andreas Jeromin, Atlantic Biomarkers, LLC, Alachua, FL 32615, USA; andreasjeromin@gmail.com

Oskar Hansson Clinical Memory Research Unit, Department of Clinical Sciences, Lund University, Lund 22184, Sweden; Eli Lilly and Company, Indianapolis, IN, USA; oskar.hansson@med.lu.se

Shorena Janelidze, Clinical Memory Research Unit, Department of Clinical Sciences, Lund University, Lund 22184, Sweden; shorena.janelidze@med.lu.se

Corresponding Author

Divya Bali, Clinical Memory Research Unit, Department of Clinical Sciences Malmö, Lund University, Sölvegatan 19, BMC B11, 221 84 Lund, Sweden; divya.bali@med.lu.se, phone +46 46-2229667.

Shorena Janelidze, Clinical Memory Research Unit, Department of Clinical Sciences Malmö, Lund University, Sölvegatan 19, BMC B11, 221 84 Lund, Sweden; shorena.janelidze@med.lu.se, phone +46 46-2229667.

Supplementary Table 1

	AUC	95% CI	p-value
**ADNC**			
p-Tau217$_{ALZpath}$	0.75	(0.63,0.87)	Reference
p-Tau181$_{Lilly}$	0.76	(0.65,0.88)	0.72
p-Tau217$_{Lilly}$	0.87	(0.78,0.96)	**0.021**
**BRAAK**			
p-Tau217$_{ALZpath}$	0.74	(0.61,0.87)	Reference
p-Tau181$_{Lilly}$	0.75	(0.61,0.88)	0.88
p-Tau217$_{Lilly}$	0.82	(0.70,0.94)	**0.021**
**CERAD**			
p-Tau217$_{ALZpath}$	0.78	(0.66,0.89)	Reference
p-Tau181$_{Lilly}$	0.79	(0.68,0.90)	0.85
p-Tau217$_{Lilly}$	0.89	(0.80,0.97)	**0.024**

Receiver operating characteristics (ROC) curve analysis for predicting ADNC, Braak, and CERAD classification. The DeLong test was used to compare area under the curve (AUC) of two ROC curves.
Abbreviations: ADNC, Alzheimer´s disease neuropathologic change; CI, confidence interval; CERAD, Consortium to Establish a Registry for Alzheimer´s Disease; p-Tau, phosphorylated tau
